# Humanization of a strategic CD3 epitope enables evaluation of clinical T-cell engagers in a fully immunocompetent in vivo model

**DOI:** 10.1038/s41598-022-06953-7

**Published:** 2022-03-03

**Authors:** Julie A. Zorn, Matthew L. Wheeler, Ralston M. Barnes, Jim Kaberna, Winse Morishige, Marek Harris, Richard Y.-C. Huang, Jack Lohre, Yu Ching Chang, Bryant Chau, Kathleen Powers, Ian Schindler, Naveen Neradugomma, Winston Thomas, Xiaoyun Liao, Yinhan Zhou, Sean M. West, Feng Wang, Srikanth Kotapati, Guodong Chen, Sayumi Yamazoe, Anastasia Kosenko, Gavin Dollinger, Tim Sproul, Arvind Rajpal, Pavel Strop

**Affiliations:** 1 Discovery Biotherapeutics, Bristol Myers Squibb, Redwood City, 94063 USA; 2 Immuno Oncology Discovery, Bristol Myers Squibb, Redwood City, 94063 USA; 3 Pharmaceutical Candidate Optimization, Bristol Myers Squibb, Lawrenceville, 08543 USA; 4 In Vivo Pharmacology, Bristol Myers Squibb, Redwood City, 94063 USA; 5 Analytical Development & Attribute Sciences, Biologics Development, Bristol Myers Squibb, New Brunswick, 08903 USA; 6 Genentech, South San Francisco, 94080 USA; 7 Biologics Discovery, Tallac Therapeutics, Burlingame, 94010 USA; 8 Translational Research, Bristol Myers Squibb, Redwood City, 94063 USA

**Keywords:** Cancer models, Cancer therapy, Haematological cancer

## Abstract

T-cell engagers (TCEs) are a growing class of biotherapeutics being investigated in the clinic for treatment of a variety of hematological and solid tumor indications. However, preclinical evaluation of TCEs in vivo has been mostly limited to xenograft tumor models in human T-cell reconstituted immunodeficient mice, which have a number of limitations. To explore the efficacy of human TCEs in fully immunocompetent hosts, we developed a knock-in mouse model (hCD3E-epi) in which a 5-residue N-terminal fragment of murine CD3-epsilon was replaced with an 11-residue stretch from the human sequence that encodes for a common epitope recognized by anti-human CD3E antibodies in the clinic. T cells from hCD3E-epi mice underwent normal thymic development and could be efficiently activated upon crosslinking of the T-cell receptor with anti-human CD3E antibodies in vitro. Furthermore, a TCE targeting human CD3E and murine CD20 induced robust T-cell redirected killing of murine CD20-positive B cells in ex vivo hCD3E-epi splenocyte cultures, and also depleted nearly 100% of peripheral B cells for up to 7 days following in vivo administration. These results highlight the utility of this novel mouse model for exploring the efficacy of human TCEs in vivo, and suggest a useful tool for evaluating TCEs in combination with immuno-oncology/non-immuno-oncology agents against heme and solid tumor targets in hosts with a fully intact immune system.

## Introduction

Bispecific antibodies are gaining prominence as an effective therapeutic modality in oncology^1^. In particular, T-cell engagers (TCEs) are showing promising efficacy in the clinic targeting hematological malignancies^2,3^. TCEs are bispecific molecules composed of a target antigen arm and a TCR/CD3 binding arm that redirects T-cell killing activity to cancer cells expressing an antigen of interest^4^.

**Figure 1:**
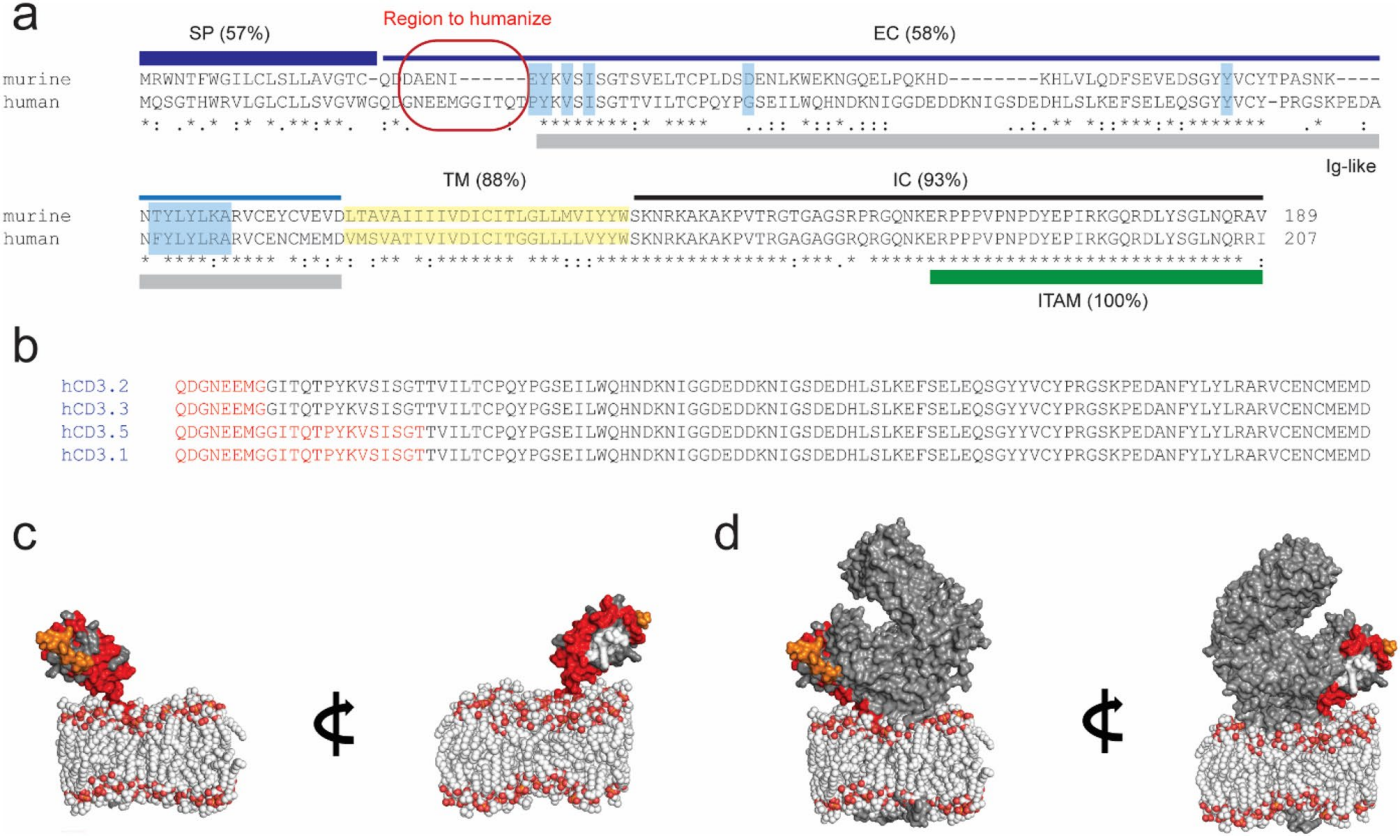
Structural rationalization for the hCD3E-epi mouse model. (**a**) Alignment of mouse and human CD3E to inform the design of the mouse model. (*SP* signal peptide, *EC* extracellular domain, *TM* transmembrane domain, *IC* intracellular domain.) Residues that are involved in binding to human CD3δ or human CD3γ are highlighted (blue). (**b**) HDX data for the anti-human CD3 molecules (red) mapped onto the sequence of human CD3E. (**c**) Structural model of human  CD3E alone in the membrane. Colors are N-terminal epitope that we replaced (orange), other human-cyno identical residues (red), human insertion in CD3 (white). (**d**) Human CD3E/D in complex with the TCR in the membrane. Only one CD3E (epsilon in complex with delta) in the TCR complex is shown, but the other (CD3 epsilon-gamma complex) looks similar. Models were generated using PBD IDs 6JXR and 2MLR.

The TCR complex on T cells is composed of the either the α/β or γ/δ T cell receptor in association with the CD3ε/γ, CD3ε/δ, and CD3ζ/ζ dimers in a 1:1:1:1 ratio^5^. Many TCEs currently in the clinic target CD3, and more specifically the CD3ε chain to promote productive T-cell activation^4,6^. The CD3ε chain consists of an N-terminal signal sequence, the extracellular domain, which is predominantly characterized by an Ig-like fold, a single-pass transmembrane domain, and an intracellular domain with a conserved ITAM motif (Fig. 1a). The sequence identity between the extracellular domains of human and murine CD3ε (hCD3E and mCD3E, respectively) is relatively low at 58%, which has limited the identification of anti-CD3 antibodies that are human/murine cross-reactive (Fig. 1a). This restricts the in vivo models that are available for testing TCEs in a preclinical setting.

Historically, TCEs have often been evaluated using xenograft models in immunodeficient mice. In this model, the mice are transfused with donor-derived human immune cells, implanted with human tumor cells, and treated with TCE therapeutic molecules. While having the advantage of testing fully human antibodies, the reconstituted immune system in this model does not resemble an intact mouse, is known to develop alloreactivity to implanted tumors, results in donor-to-donor variations, and restricts evaluation of off-target toxicities associated with treatment.

More recently, efforts have focused on evaluating TCEs in syngeneic models. In particular, murine bispecific technologies have been developed to allow surrogate TCEs to be evaluated in immunocompetent  wild-type mice^7,8^. While this addresses many of the limitations associated with xenograft models, the CD3 arm used for the molecules targets murine CD3 and may not accurately recapitulate the activity of anti-human CD3 bispecifics.

Finally, to evaluate the in vivo efficacy of anti-human CD3 TCEs in an immunocompetent mouse, transgenic models expressing human CD3 components have been developed. However, in these models, complete replacement of the entire CD3 complex or replacement with the CD3E chain alone result in deficiencies in T-cell frequencies or function^9,10^. More recent developments have focused on human/murine chimeras, replacing targeted exons within the murine *Cd3e* locus^11,12^. While these models are more promising, the chimeras introduce mutations in murine CD3E at residues important for interactions with CD3G and CD3D.

Thus, we sought to develop a preclinical syngeneic model that was both generalizable to a broad class of TCEs and minimalistic in modifications to the murine TCR/CD3 complex. Here, we generated a transgenic mouse where we introduced a targeted replacement of an N-terminal DAENI motif in murine CD3e with a GNEEMGGITQT motif from hCD3E. We identified this region on hCD3E as the target of many anti-human CD3 arms from TCEs currently in the clinic. Importantly, this modest replacement in the design of the hCD3E epitope (hCD3E-epi) knock-in mouse does not impact interactions within the TCR complex. Consequently, we do not observe deficiencies in T-cell frequency or function that have been noted with other models. Further, we showed the utility of the model by demonstrating in vivo efficacy with an anti-human CD3/CD20 TCE in a B-cell depletion study.

## Results

### Structural rationale for hCD3E-epi knock-in mouse design

To aid in the development of a syngeneic model that would enable the evaluation of a broad class of TCEs, we characterized the binding properties of a panel of anti-CD3 arms for TCEs currently in the clinic. This panel of anti-human CD3 antibodies includes not only some of the common cynomolgus monkey (cyno) cross-reactive SP34-derived clones, but also unique clones as well (Table S1)^13,14^.

First, we used surface plasmon resonance (SPR) to confirm the binding properties of our panel of anti-CD3 Fabs to purified human, cyno, and murine Fc-tagged CD3E/D heterodimers (Table 1 and Fig. S2). We verified that the anti-human CD3 Fabs in our panel, hCD3.1–hCD3.5 as well as SP34, bound potently to cyno and hCD3E/D, but not to mCD3E/D. Additionally, we used the murine surrogate anti-CD3 clone, 2C11, to confirm binding to mCD3E/D, but not cyno or human CD3E/D (Table 1 and Fig. S3)^15^. Thus, this in vitro characterization supported the inability of selected clinical anti-human CD3 variants to bind to mCD3E.

**Table 1:** Characterization of anti-CD3 Fabs binding to different CD3E/D variants by SPR.

Fab	Human CD3E/D	Cyno CD3E/D	Murine CD3E/D	Chimeric human/ murine CD3E/D
	K_D_ (nM)	K_D_ (nM)	K_D_ (nM)	K_D_ (nM)
hCD3.1	6.3 ± 0.18	6.5 ± 0.11	NB	8.6 ± 0.53
hCD3.2	3.1 ± 0.35	3.8 ± 0.61	NB	4.2 ± 0.22
hCD3.3	0.77 ± 0.13	0.91 ± 0.17	NB	0.81 ± 0.06
hCD3.4	2.7 ± 0.45	2.7 ± 0.02	NB	3.1 ± 0.23
hCD3.5	4.1 ± 0.44	5.0 ± 0.5	NB	4.4 ± 0.27
SP34	6.6 ± 0.46	7.5 ± 0.06	NB	7.3 ± 0.18
2C11	NB	NB	22.0 ± 1.4	250 ± 16

*NB* no binding.

To inform the humanization strategy of mCD3E, we then determined the epitopes for our panel of anti-CD3 binding arms using hydrogen/deuterium exchange mass spectrometry (HDX-MS) (Fig. 1b). HDX reveals changes in solvent accessibility and/or hydrogen bonding induced by complex formation on protein backbone amides. Enzymatic digestion of the Fc-tagged hCD3E/D heterodimeric protein alone with pepsin under conditions used in the HDX-MS workflow provided 98.1% and 97.6% sequence coverage of the E- and D-subunits, respectively, giving confidence to complete epitope characterization using this approach (Fig. S4). We next performed HDX on the complexes between our panel of anti-human CD3 containing bispecifics and the hCD3E/D heterodimer. The peptide-level deuterium uptakes for these complexes were evaluated relative to hCD3E/D alone to reveal regions of the protein that were protected (Fig. S5). The epitopes for all the anti-human CD3 containing bispecifics in our panel mapped to the N-terminus (Fig. 1b).

To validate our engineering strategy of incorporating the anti-human CD3 epitope into mCD3E, we purified a recombinant, chimeric human/murine CD3E protein where we replaced a 5-residue N-terminal stretch in mCD3E with a 11-residue stretch from hCD3E and expressed this as a h/mCD3E—mCD3D Fc-tagged heterodimer. We demonstrated similar binding affinities of our anti-human CD3 Fabs to this chimera relative to hCD3E by SPR (Table 1). These comparable affinities indicate that this modest change in mCD3E was sufficient to accommodate binding of the anti-human CD3 variants. Notably, 2C11, which has an epitope on mCD3E that includes both the N-terminus as well as residues adjacent to the N-terminus, still bound to this chimera, but with reduced affinity (Table 1).

We next sought to understand if there were additional surface accessible epitopes on hCD3E that could be targeted by novel anti-human CD3 antibodies not represented in our panel, or if this minimal replacement of the N-terminus in mCD3E would be sufficient to study a broad class of TCEs. We focused on residues conserved between human and cyno CD3E to enable rapid clinical development. In evaluating the structure of the hCD3E subunit alone, there appear to be multiple regions on the surface of the protein that would be tractable for binding a human/cyno cross-reactive anti-CD3 antibody (Fig. 1c). However, a recent report on the cryo-EM structure of the TCR/CD3 complex reveals that most of these regions on CD3E become buried in the complex and are not accessible to an antibody^5^ (Fig. 1d).

In the context of the TCR/CD3E/D complex, only three regions appear to be human/ cyno cross reactive and surface exposed: (1) a membrane proximal patch, (2) a region adjacent to the N-terminus, and (3) the N-terminus (Fig. 1d). The first human/ cyno cross-reactive region closest to the membrane would likely be inaccessible to anti-CD3 antibodies. The second region proximal to the N-terminus is a relatively small area that would be difficult to target with a high affinity anti-CD3E binder. Additionally, this second area is adjacent to an insertion in the hCD3E protein that makes the local surface different from the cyno CD3E protein and thus, more challenging to identify antibodies that are cross-reactive that target this epitope (Fig. 1d and Fig. S3). This leaves the N-terminus as the most tractable epitope for targeting by anti-CD3E antibodies that are human/cyno cross-reactive.

### Generation of immunocompetent mice with a humanized CD3E strategic epitope

**Figure 2:**
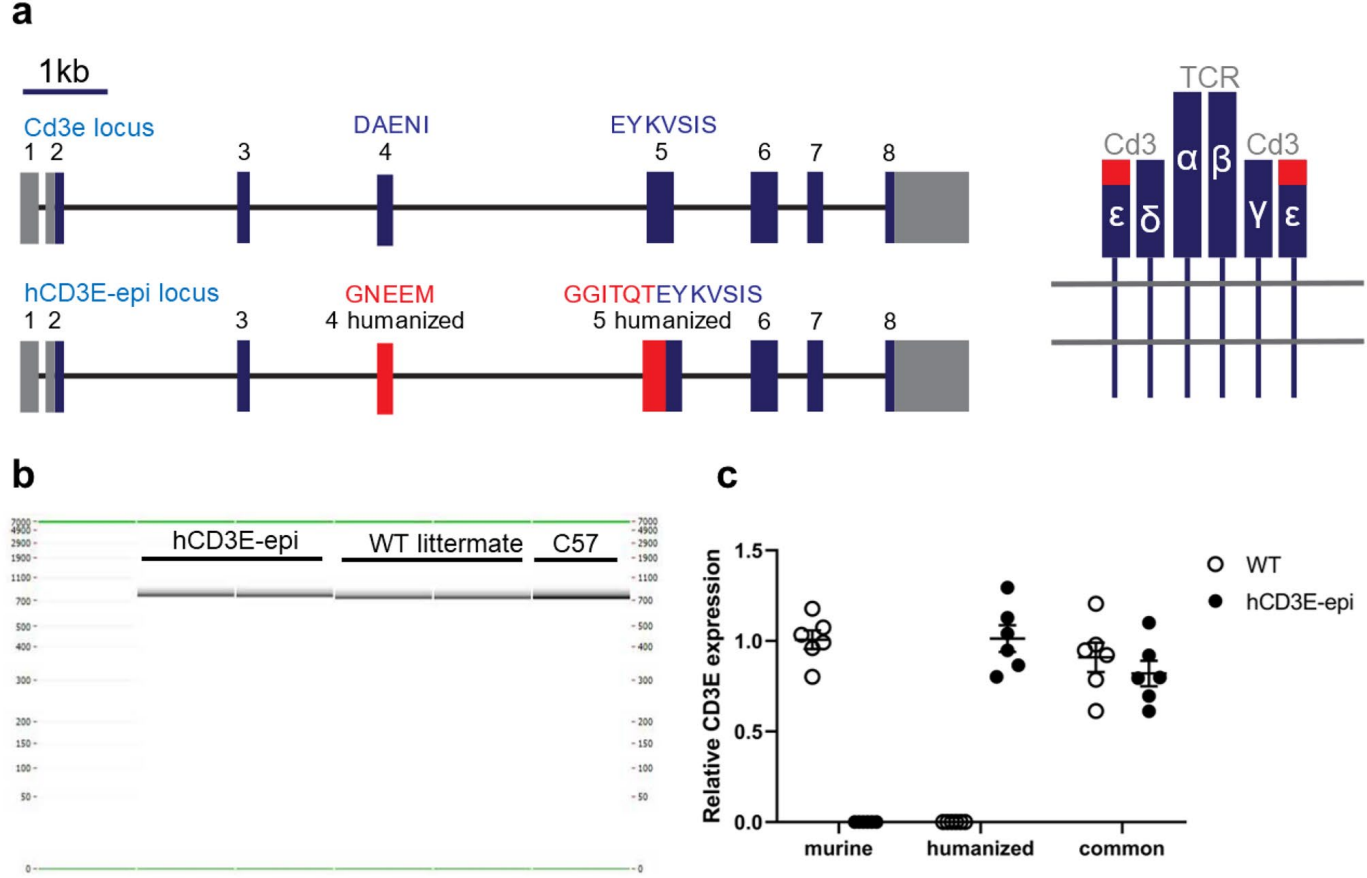
Schematic and expression analysis of the tactical Cd3e humanization strategy. (**a**) Epitope humanization strategy of the murine *Cd3e* locus with UTR (grey), exons (blue), and humanized exons (red). Amino acid residues for exon 4 and exon 5 are shown in the schematic alongside a diagram of the Cd3-TCR complex. (**b**) Endpoint RT-PCR across the humanization region in WT and homozygous hCD3E-epi mice showing a single transcript without alternative splicing. (**c**) qRT-PCR on thymic T cells from 7-week old mice showing relative expression in WT and homozygous hCD3E-epi mice normalized to murine Actb. Each point represents an individual mouse. Results are shown as mean ± SEM; WT vs hCD3E-epi common assay expression: ns, *p* > 0.05.

Following identification of a high value epitope for CD3 biologics, we designed a strategy to humanize the murine *Cd3e* locus in an immunocompetent background. The corresponding murine epitope was modified through a germline replacement of exon 4 and exon 5 by homologous recombination in C57BL/6 (B6) mouse embryonic stem (ES) cells with a targeting construct engineered to introduce the human germline sequence. This enabled creation of the strategic humanization by replacing the DAENI motif with a GNEEMGGITQT motif (Fig. 2a). Bioinformatic evaluation of ENCODE data for *Cd3e* and *CD3E* germline sequence did not identify any predicted regulatory motifs that would be removed or transferred through the germline targeting event (data not shown) indicating the targeting strategy should be low risk to alter physiological expression or expression pattern in the humanized mice. Following germline transmission and breeding to B6 mice the humanized *Cd3e* mice were born at normal Mendelian ratios and then bred to homozygosity (hCD3E-epi).

The hCD3E-epi mice were first evaluated for genomic integrity following humanization. Splenocytes from the hCD3E-epi mice were sequenced by Targeted Locus Amplification (TLA) to enable complete gene sequencing following the humanization^16^. Data from TLA confirmed the correct targeting event and established that no unexpected genomic alteration occurred in or around the *Cd3e* locus itself or within neighboring genes, including genetically linked *Cd3d* and *Cd3g* genes (Fig. S1A).

Following genomic DNA confirmation, the hCD3E-epi mice were evaluated for transcript functionality. Endpoint RT-PCR assays were set up across the humanization region for both wild-type (WT) and hCD3E-epi mice. Data resulted in only a single, expected band for both genotypes (Fig. 2b). Amplicons were then sequenced and resulted in the designed humanized sequence across the exon boundaries (Fig. S1B). The results confirmed the strategic humanization generated the expected transcript and did not result in cryptic or impaired splicing. To determine if the humanization strategy altered expression levels or patterns, we then isolated T cells from the thymus of 7-week-old mice. T cells were isolated in similar quantities (data not shown) and quantitative PCR was performed with WT and hCD3E-epi specific assays as well as a common assay for comparison between the hCD3E-epi and WT strains. The assays distinguished between the different strains, and the results from the common assay confirmed similar relative *Cd3e* expression in WT and hCD3E-epi mice (Fig. 2c; unpaired t-test, *p* = 0.43).

**Figure 3:**
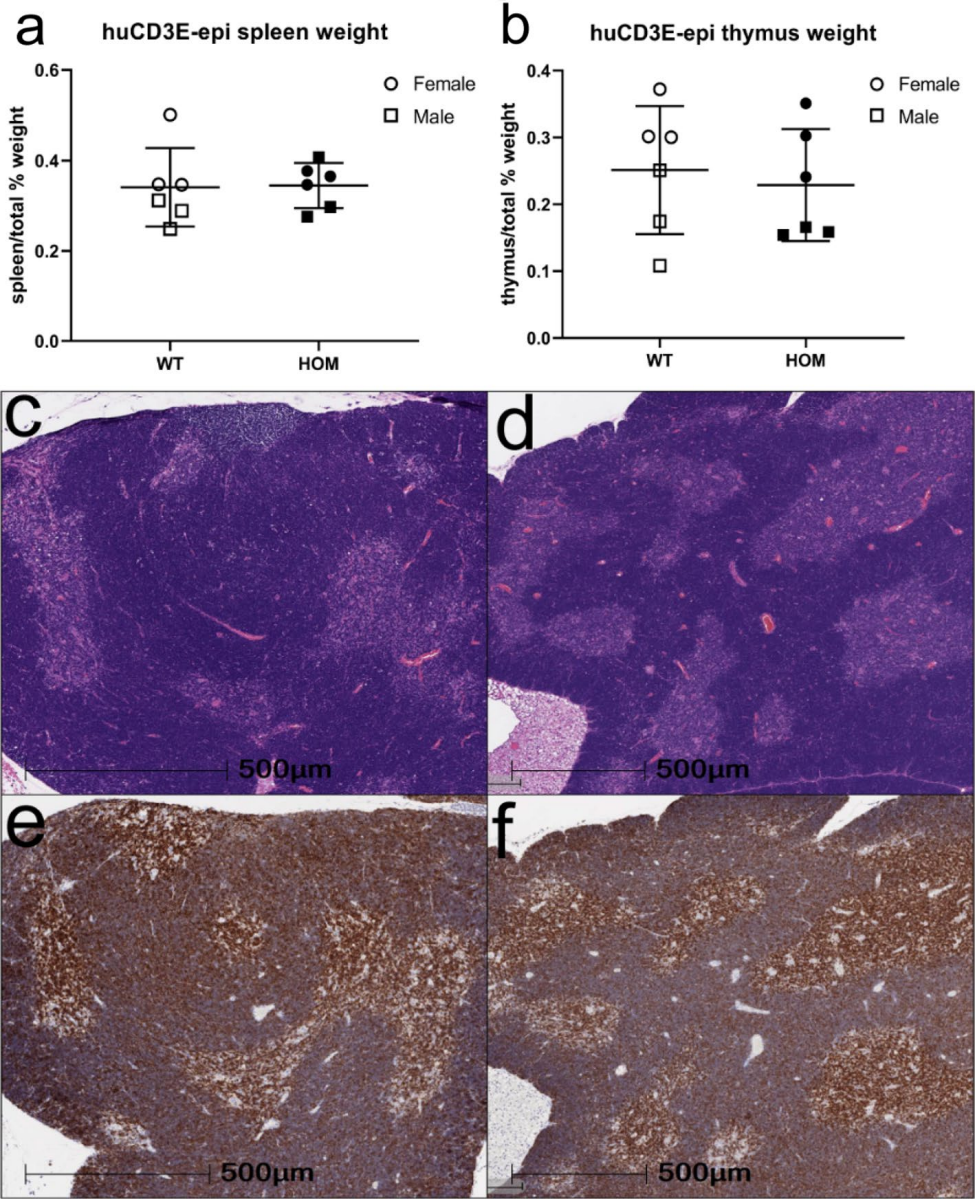
Characterization of thymus and spleens in hCD3E-epi mice. Spleen (**a**) and thymus (**b**) were weighed across WT littermate and hCD3E-epi 7-week old mice (n = 6). Weights are graphed as a percentage of total body weight. Each point represents an individual mouse. Results are shown as mean ± SD, ns, *p* > 0.05. Representative images of hCD3E-epi (**c**,**e**) and WT littermates (**d**,**f**) for both H&E and IHC staining. Similar histology and CD3E staining pattern were observed in thymi of hCD3E-epi and WT mice. (**c**,**d**) H&E staining (**e**,**f**) CD3E staining.

Previously generated human CD3 transgenic models have resulted in altered T-cell function and a range of phenotypes, including thymic dysplasia^9,10^. hCD3E-epi mice were designed to maintain native CD3-complex and function to support T-cell activation and function. Phenotypic evaluation of the hCD3E-epi mice began with an initial evaluation of relative thymus weight in 7-week-old mice to gain insight into T-cell function and development. Both spleens and thymi were weighed and normalized for both male and female WT and hCD3E-epi mice. Results indicated no significant difference between genotypes when comparing the weight of either spleens or thymi across WT and hCD3E-epi mice (Fig. 3a,b) (*n* = 6/genotype; unpaired t-test, *p* = 0.92 (a), *p* = 0.68 (b)). This gross analysis indicates the humanization does not result in thymic dysplasia and likely supports normal T-cell development and activation. Histological analysis was also performed on WT and hCD3E-epi mice. The histological architecture in the thymi of hCD3E-epi and WT mice were very similar. Cortical and medullary regions were observed within each lobule along with similar CD3e staining pattern (Fig. 3c–f). No pathology was observed in hCD3E-epi mice, and normal development was observed across samples. Together, the gross and histological analysis support normal T-cell and thymic development in hCD3E-epi mice.

### Validation of hCD3E-epi mice as a physiological model to study human T cell engaging bispecific antibodies in vitro and in vivo

**Figure 4:**
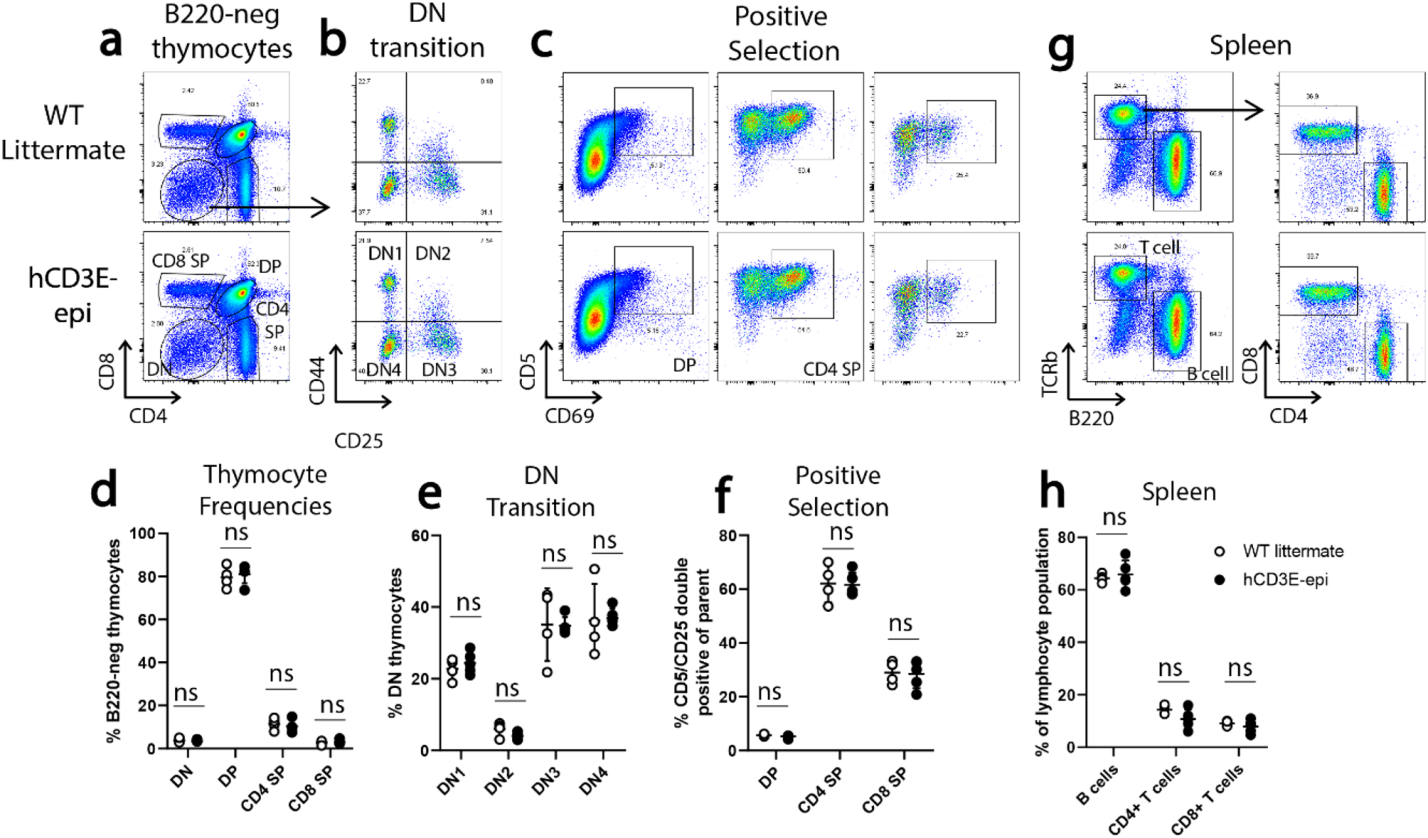
Normal T cell development in hCD3E-epi mice. (**a**–**f**) Representative flow cytometric staining and quantification of thymocytes from WT littermate controls (top row) and hCD3E-epi mice (bottom row). (**a**) Thymocyte frequencies defined by CD4 and CD8 surface staining to identify single positive (SP), double positive (DP), and double negative thymocytes (DN). Samples are quantified in (**d**) as a percentage of live B220-negative thymocytes. (**b**) Representative staining to depict transition through double DN1-4 stages of thymocyte development, defined by surface CD44 and CD25 staining. Samples are quantified in (**e**) as a percentage of total live DN thymocytes (**c**) Representative staining to depict thymocytes undergoing positive selection at the double positive, CD4 SP, and CD8 SP stages (left to right), defined by surface expression of CD5 and CD69 activation markers. Data are quantified in (**f**) as the percentage of CD5-positive and CD69-positive withing the indicated parent population. (**g**) Representative flow cytometric staining and quantification (**h**) of B cells and CD4/8 T cells in spleens, calculated as a percentage of the total live lymphocyte population. Each symbol represents a single mouse. WT littermate (n = 4 open circles) and hCD3E-epi (n = 5 filled circles). Data are representative of two individual experiments. No significant differences in thymocyte/lymphocyte frequencies were observed using student unpaired T test (ns = not significant, p > 0.05). Error bars represent mean + /- SEM.

TCR signaling plays a critical role at all stages of T cell development and selection in the thymus^17^. To ensure that humanization of the *Cd3e* locus did not impact T cell development or functional maturation of T cells, we first analyzed the frequency of T cells at different stages of positive and negative selection in the thymus (Fig. 4a–e). No significant differences were observed in frequencies of CD4/CD8 double negative, single positive, or double positive thymocytes between WT and hCD3E-epi mice (Fig. 4a,d). Furthermore, frequencies of thymocytes at different stages of double negative transition (Fig. 4b,e) and undergoing positive selection (Fig. 4c,f) were unaffected by humanization of the *Cd3e* locus. Similarly, no differences were observed in the frequency of CD4 and CD8 T cells in the spleen, indicating that T cell development and thymic emigration were unaffected by *Cd3e* humanization (Fig. 4g,h).

**Figure 5:**
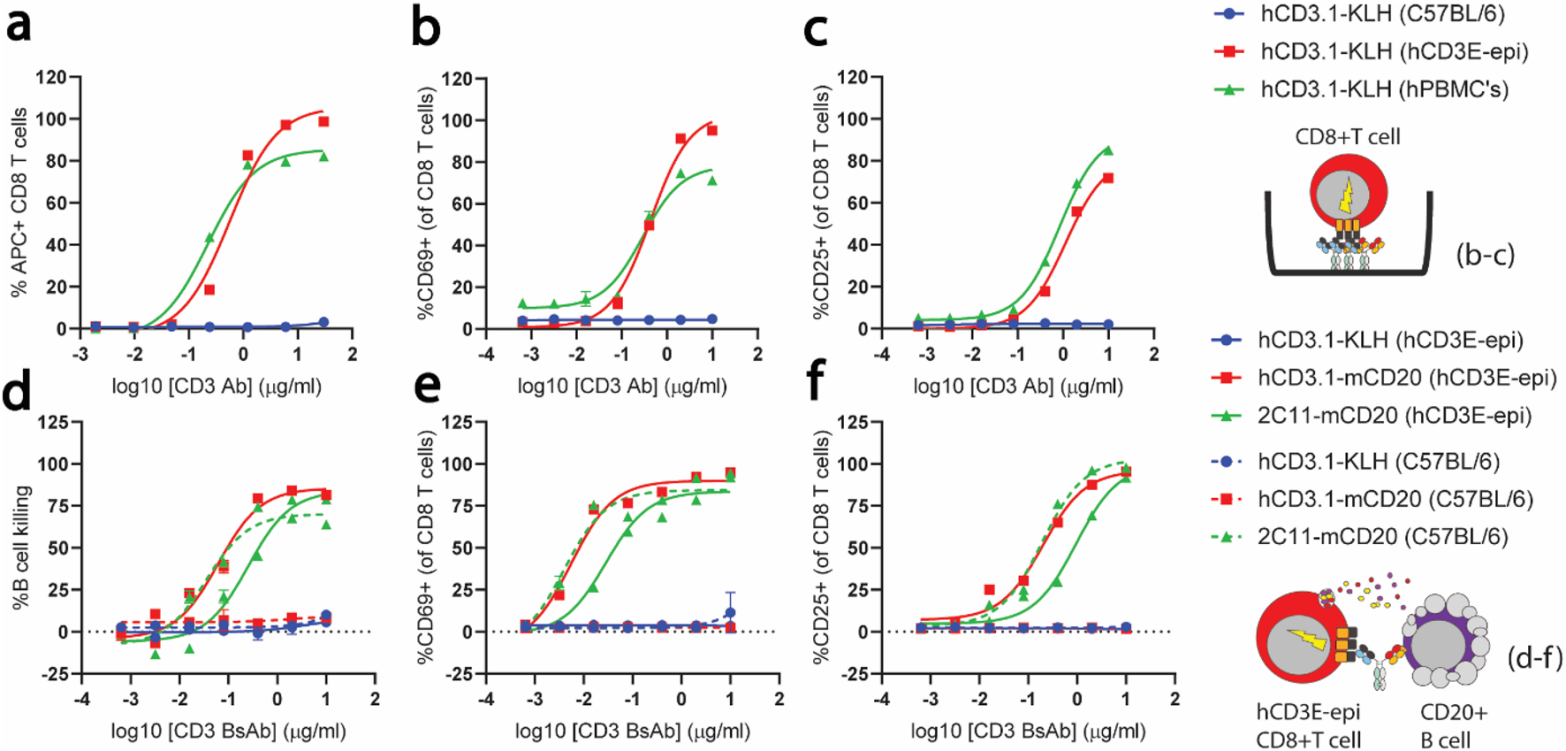
Normal activation and cytotoxic function of T cells from hCD3E-epi mice using anti-human and mouse CD3-bispecific antibodies. (**a**) APC-conjugated anti hCD3.1-KLH antibody was titrated onto WT splenocytes (blue circles) hCD3E-epi splenocytes (red squares), and human PBMC’s (green triangle), and binding to the surface of CD8 T cells was quantified by calculating the mean fluorescence intensity of surface stain at indicated concentrations of antibody by flow cytometry. (**b**,**c**) WT splenocytes, hCD3E-epi splenocytes, and human PBMC’s were cultured in the presence of indicated concentrations of plate-bound anti hCD3.1-KLH antibody, and T cell activation was calculated as the percentage of CD8 + T cells that upregulated surface CD69 (**b**) or CD25 (**c**) by flow cytometry after 72 h of stimulation. Data are representative of replicate stimulations from n = 3 pooled spleens, or a single hPBMC donor, and are representative of 2 individual experiments. (**d**–**f**) hCD3E-epi and WT T cell redirected killing of murine B cells and T cell activation upon incubation of splenocytes with CD3-CD20 BsAbs. (**d**) In vitro B cell killing was extrapolated from the percent of live CD19 + B cells remaining in splenocyte culture from WT (dashed lines) and hCD3E-epi (solid lines) mice after 72 h of stimulation with indicated concentrations of hCD3.1-mCD20, 2C11-mCD20, hCD3.1-KLH, and 2C11-KLH bispecific antibodies. T cell activation from the same splenocyte cultures was measured by flow cytometry as the percentage of CD8 + T cells that upregulated CD69 (**e**) or CD25 (**f**) at 72 h following incubation with indicated bispecific antibodies. All data (**a**–**f**) are representative of duplicate stainings/in vitro stimulations from n = 3 pooled spleens, or a single hPBMC donor, and are representative of 2 individual experiments. Curves were fit using three-parameter nonlinear regression.

Consistent with SPR results, anti-human CD3 (hCD3.1-KLH) bound to the humanized CD3E on the surface of hCD3E-epi T cells and human peripheral blood T cells with similar affinity, but did not bind to WT C57BL/6T cells as expected (Fig. 5a). To ensure normal functionality of T cells from hCD3E-epi mice, T cell activation with a plate-bound anti-human CD3 antibody (hCD3.1-KLH) was monitored after 72 h of stimulation. Similar dose-dependent upregulation of T cell activation markers (CD69 and CD25) was observed on CD4 (not shown) and CD8 T cells from hCD3E-epi splenocytes and human PBMC’s, but not WT mice stimulated with plate-bound anti-human CD3 (Fig. 5b,c), indicating that anti-human CD3 antibodies are able to effectively bind and crosslink the T cell receptor to activate hCD3E-epi T cells in vitro.

To explore the utility of this mouse model for preclinical assessment of human CD3E-targeted biologic therapies, we tested the ability of hCD3E-epi T cells to mediate cytotoxic killing of target cells following treatment with a T cell-engaging CD3-bispecific antibody (TCE). Incubation of WT and hCD3E-epi total splenocytes with a bispecific T cell engager targeting human CD3 and murine CD20 (hCD3.1-mCD20), resulted in efficient T cell-redirected killing of B cells in hCD3E-epi but not WT spleens after 48 h of in vitro treatment (Fig. 5d). In contrast a CD3-bispecific targeting murine CD3 and murine CD20 (m2C11-mCD20) was able to deplete B cells in both hCD3E-epi and WT splenocyte cultures (Fig. 5d). T cell activation kinetics (CD69, CD25 on CD8-pos T cell) also mirrored B cell killing in this in vitro setup (Fig. 5e,f). These results indicate that the humanized CD3E epitope is distinct from the epitope recognized by a widely used commercial anti-mouse CD3E antibody, and furthermore, suggests that humanization has minimal effects on the native murine CD3 complex.

**Figure 6:**
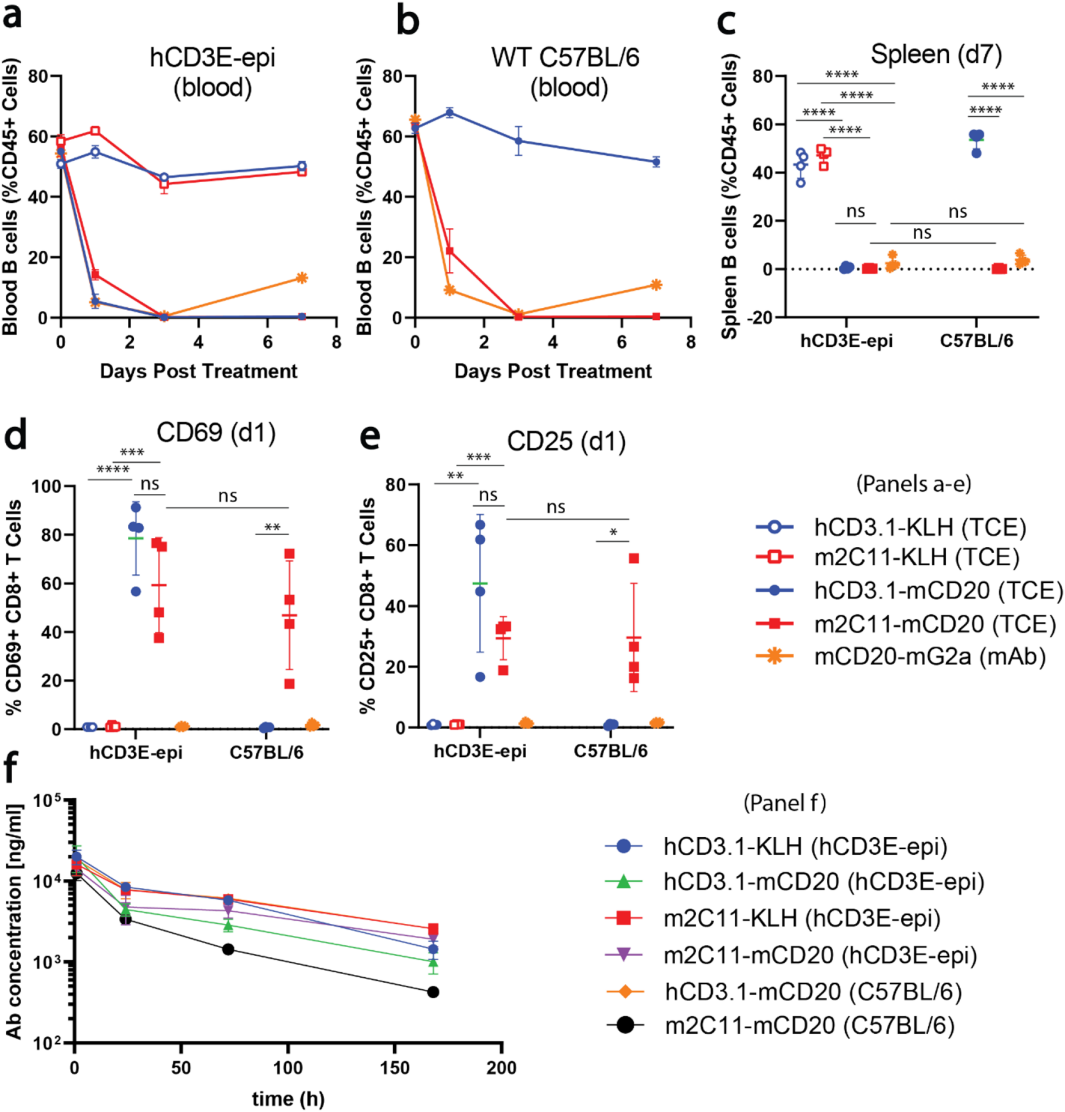
In vivo B cell depletion and T cell activation following intravenous CD3-CD20 bispecific antibody treatment. (**a**,**b**) CD19 + B cell frequencies, as a percentage of total CD45 + leukocytes, were measured in the peripheral blood of hCD3E-epi (**a**) and WT C57BL/6 mice on day 0 (pre-dose), 1, 3 and 7, and in the spleen on day 7 (**c**), following intravenous treatment with1 mg/kg of the indicated CD3-bispecific antibodies, or 10 mg/kg of mCD20-mG2a. Peripheral T cell activation was measured as a percentage of total blood CD8 + T cells that upregulated CD69 (**d**) or CD25 (**e**) in hCD3E-epi and WT C57BL/6 mice on day 1 post treatment with the indicated antibodies. (**f**) Peripheral exposure of indicated the CD3-bispecific antibodies was measured in the serum hCD3E-epi and WT C57BL/6 mice at 1, 24, 72, and 168 h following IV treatment. Each point represents an individual mouse (n = 4/group). Error bars represent ± SEM. P values were calculated using unpaired student T-test. NS (not significant *p* > 0.05), *p ≤ 0.05, ** p ≤ 0.01, *** p ≤ 0.001, ****p ≤ 0.0001. Data are representative of 2 independent experiments.

To investigate in vivo relevance of this mouse model, we treated WT and hCD3E-epi mice intravenously (IV) with the same TCE’s used in the in vitro killing assay and monitored peripheral B cell depletion over 7 days. A single IV dose of hCD3.1-mCD20 was sufficient to deplete nearly 100% of CD19-positive B cells from the peripheral blood (day 1–7 post treatment) (Fig. 6a,b) and spleen (day 7 post treatment) (Fig. 6c) of hCD3E-epi mice, but not WT mice, as expected. B cell depletion in hCD3E-epi mice was also accompanied by rapid activation of peripheral T cells, as measured by upregulation of surface CD69 and CD25 on blood CD8 T cells 24 h post-treatment (Fig. 6d,e). Both B cell depletion and T cell activation in hCD3E-epi mice required dual engagement of CD3 and CD20 since control antibodies targeting CD3 and an irrelevant targeting arm (KLH) had no obvious effect on peripheral cellularity or immune activation state (Fig. 6a,c,d,e). Importantly, similar levels of B cell depletion and peripheral T cell activation were observed following treatment of both WT and hCD3E-epi mice with m2C11-mCD20, as well as with an anti-mouse monoclonal CD20 depleting antibody (mCD20-IgG2a) (Fig. 6a–e). Serum exposure analysis of the treatment antibodies demonstrated clearance rates consistent with native mouse IgGs (Fig. 6f). We did observe a modest reduction in peripheral exposure of m2C11-mCD20 in WT mice compared to hCD3E-epi mice (Fig. 6f black filled circles vs. purple filled triangles), which likely reflects the increased affinity of m2C11 for the native murine CD3E over chimeric hCD3E-epi. These differences in exposure did not impact the treatment response, as we saw similar levels of peripheral B cell depletion and T cell activation in WT and hCD3E-epi mice treated with m2C11-CD20 TCEs (Fig. 6a–e). These results indicate that hCD3E-epi mice are suitable for studying both murine and human CD3 targeted therapies, and also retain normal antibody-dependent-cellular-cytotoxicity function.

## Discussion

In this study, we describe the design and generation of a hCD3E epitope knock-in mouse model. To inform on a strategy to humanize murine CD3E, we first characterized the binding epitope for a small panel of anti-human CD3E arms from TCEs currently in the clinic. The epitope for all of these antibodies mapped to an N-terminal region of hCD3E that is conserved in cyno CD3. With this knowledge, we engineered minimal changes in the murine *Cd3e* locus with replacement of just a 5-residue motif in mCD3E with an 11-residue motif from hCD3E. These transgenic mice displayed normal T cell development, function, and frequencies. Finally, we were able to demonstrate the utility of this model in a preclinical setting through in vitro T cell killing and activation assays, as well as through B cell depletion in mice treated with a hCD3/CD20 TCE. In conclusion, we show that making this targeted, minimal replacement in mCD3E resulted in a syngeneic model where T cell function is not impaired, which enables studying TCEs that have anti-human CD3E arms.

Of note, our panel of anti-human CD3 clinical antibodies did all map to the N-terminus of human CD3E, likely influenced by a common desire to obtain a human/ cyno cross-reactive antibody that would be surface accessible in the context of the TCR/CD3 complex. However, other strategic epitopes on CD3 have been reported. For example, OKT3 does map to a region adjacent to the N-terminus of hCD3E (Fig. S2), but consistent with our structural analysis, OKT3 is reported to have a weak affinity for hCD3E^18^. Further, others have reported targeting distinct sites on the TCR/CD3 complex^19^. These studies suggest that strategic selection of alternative epitopes may influence signaling cascades that affect T cell activation and ultimately TCE activity^19^. To characterize molecules binding hCD3E epitopes not targeting the N-terminus in a preclinical setting, additional strategies for model development would need to be considered. However, for many TCEs, our model provides a way to assess T cell activity and therapeutic effects with native TCR characteristics.

TCEs represent a growing class of bispecific molecules that are showing promising results in cancer immunotherapy. Thus far, two TCEs have been clinically approved—catumaxomab, an EPCAM/CD3 bispecific, for malignant ascites and blinatumomab, a CD19/CD3 bispecific, for acute lymphoblastic leukemia (ALL)^20^, which has sparked efforts to advance additional TCEs targeting both hematologic and solid tumor indications. In the clinic, TCEs have been shown to be particularly effective in treating hematologic tumors but have been met with significant challenges in the solid tumor setting, including limited efficacy and off-tumor on-target toxicities.

Despite signs in the preclinical setting that TCEs demonstrated promising efficacy and reasonable therapeutic windows against solid tumors, there is limited clinical success. In part, this dichotomy could be due to the restricted models available to evaluate TCEs in vivo. Typically, TCEs have been evaluated with xenograft models in immunodeficient mice. Unfortunately, these models do not recapitulate the immune system of an intact mouse and restrict the ability to evaluate on-target toxicities. While utilizing surrogate TCEs in syngeneic models does enable the assessment of molecules in a more relevant,  immunocompetentt setting, there is the possibility that results using the mouse bispecifics might not translate to the human setting. More recent efforts to generate transgenic mouse models with a humanized CD3 offer the most promise to evaluate TCEs, to define mechanisms that limit activity, and to identify approaches to avoid normal tissue toxicities. However, models generated to date are typically confounded by deficiencies in T cell frequencies or function^9,10^. The hCD3E-epi knock-in mouse enables characterization of both efficacy and toxicity for TCEs that have an anti-human CD3 binding arm and a murine/human cross-reactive tumor antigen arm^21^.

This novel hCD3E-epi knock-in model offers the opportunity to further evaluate and characterize TCEs in a solid tumor setting leveraging syngeneic tumor cell lines^21^. Some of the challenges associated with the discovery of effective and safe therapies in solid tumor indications include heterogenous expression of the tumor antigen, an immune suppressive tumor microenvironment, and high expression of solid tumor targets in normal tissue leading to off-tumor, on-target toxicities^22^. In this context, TCEs will need to be evaluated in combination with other immunotherapy agents to achieve potent activity^12,23^. In the hCD3E-epi mouse model, we are able to evaluate both single agent activity and combination activities to further understand the role of each of these factors, as well as ways to generate better molecules in this setting.

## Methods

### Human CD3E knock-in mice

A *Cd3e* targeting vector was constructed from genomic C57BL/6N mouse DNA (genOway). Targeting was designed to replace mouse DAENI amino acid sequence with its human counterpart GNEEMGGITQT, spanning exons 4 and 5. A *lox*P-flanked neomycin cassette was inserted in mouse intron 4. Linearized targeting vector was electroporated into C57BL/6N embryonic stem cells. G-418 resistant ES cell clones carrying the recombined locus were identified by PCR while the integrity of the human epitope sequence was confirmed by sequencing.

Recombined ES cell clones were microinjected into C57BL/6N blastocysts, giving rise to male chimeras. These male mice were crossed with C57BL/6N mice expressing Cre-recombinase to produce humanized *Cd3e* heterozygous mice, without the neomycin cassette. Heterozygous mice were validated by PCR allowing the identification of the wild-type allele and the humanized epitope allele lacking the neomycin cassette. A subset of PCR validated animals was then further characterized by DNA sequencing to fully confirm the integrity of the humanized region (data not shown). Heterozygous humanized animals were interbred to produce homozygous humanized *Cd3e* mice for study. Mendelian ratios were observed.

### Genotyping of hCD3E-epi mice

The hCD3E-epi mice were genotyped using the following primer pair: Fwd: 5’ – TGGGTTGTTATCTATTCTGTCTGGAAGACG – 3’; Rev: 5’ – TTAGCATATAGGCAGTAGACAGGGACTTGG – 3’, which produced a 325 bp band for the wild-type allele and a 398 bp band for the humanized allele. An additional forward primer, specific to the Neo selection cassette, was used in conjunction with the reverse primer listed above, to confirm deletion of the Neo cassette: Neo Fwd: 5’—ACAACAGCACCATTGTCCACTTGTCC – 3’. A 259 bp band would be produced with this primer pair only in the presence of the intact Neo cassette.

### RT-PCR for evaluation of hCD3E-epi RNA splicing

To verify proper hCd3e splicing in hCD3E-epi mice, end-point RT-PCR was performed on total RNA extracted from splenic T cells using the following primers: Fwd: 5’- CTGTCTGCGTCTGGTGCCTTCTT – 3’; Rev: 5’ – TGGGTCCACAGAAGGCGATGTCT – 3’. Spleens were extracted from 20-week old WT and hCD3E-epi mice, and single cell suspensions were prepared using gentleMACS Dissociator (Miltenyi Biotec). T cell enrichment was performed using the Pan T Cell Isolation Kit II (Miltenyi Biotec), and total RNA was then extracted using the RNeasy Mini Kit (Qiagen). Quality of the extracted RNA was verified on LabChip (PerkinElmer), and RT-PCR was performed using the SuperScript III One-Step RT-PCR Kit (Invitrogen). Resulting amplicons were analyzed on LabChip (PerkinElmer) or Sanger sequenced and confirmed against the hCD3E-epi reference sequence.

### qRT-PCR for evaluation of hCd3e and mouse endogenous Cd3e gene expression

qRT-PCR experiments were performed on total RNA extracted from thymic T cells using fluorescent probes designed specifically to the mutant and WT sequences within the humanization region, and a common sequence shared between the WT and humanized alleles spanning the exon 6 – 7 boundary. Murine Actb was used as a reference gene. Thymi were extracted from male and female 7-week old WT and hCD3E-epi mice and dissociated into single cell suspensions using gentleMACS Dissociator (Miltenyi Biotec). T cell enrichment and total RNA extraction were performed as described above. Quality of extracted RNA was verified on LabChip (PerkinElmer) and qRT-PCR was performed in triplicates using the iTaq Universal Probes One-Step Kit (Bio-Rad) on the CFX96 Touch Real-Time PCR Detection Systemin (Bio-Rad). Gene expression data were analyzed with CFX Maestro software (Bio-Rad).

### Targeted Locus Amplification (TLA) analysis of hCD3E-epi mice

Bone marrow from 20-week old hCD3E-epi mice was harvested and processed according to the bone marrow isolation protocol provided by Cergentis (Cergentis, The Netherlands). Frozen bone marrow samples were sent to Cergentis for amplification, sequencing, and bioinformatic analysis.

### Histological Analysis of hCD3E-epi mice

After mice were sacrificed, thymi from six hCD3e epi and WT littermate mice were collected, fixed in 10% neutral buffered formalin and then embedded in paraffin. 4-µm thick sections were stained with H&E and CD3e. IHC staining was performed using an automated staining system (Leica Bond RX, Leica Biosystems) with a rabbit monoclonal CD3e antibody (clone D4V8L, Cell Signaling Technology, dilution 1:100). The antibody was detected with the Leica Bond Polymer Refine detection kit (Leica Biosystems, cat# DS9800). Positive (mouse spleen) and negative (mouse heart) controls were added in IHC staining. All stained slides were reviewed by an experienced pathologist (X.L.) under light microscope.

### Assessment of T cell development in hCD3E-epi mice

Single cell suspension of spleen and thymus from WT and CD3e KI mice were prepared in RBC lysis buffer (Biolegend) using a gentle MACS tissue dissociator (Miltenyi Biotech) and resuspended at 1 × 10^7^ cells/ml in PBS (Corning) containing 2% HI-FBS (VWR), and 1 mM EDTA (Corning). 1 × 10^6^ cells were pelleted in 96 well round bottom plates and resuspended in PBS + 2%FBS + 1 mM EDTA (Flow buffer) containing Fc-block (BD), and live/dead fixable dye (Invitrogen) for 20 min. Cells were re-pelleted and resuspended in surface staining cocktail containing antibodies against mouse TCR-beta, CD8, CD4, B220, CD25, CD5, CD44, CD69 at concentrations recommended by manufacturer. Cells were washed 2 times with flow buffer and fixed with cytofix (BD) followed by 2 washes according to manufacturer’s instructions. Cells were acquired on an LSRFortessa X-20 (BD), and analyzed using Flowjo V10.6.2. Thymocytes were analyzed from the live B220-negative population, using surface CD8 and CD8 to define SP, DP, and DN thymocytes. Surface CD44 and CD25 on DN thymocytes was used to define the DN1 (CD44-pos CD25-neg), DN2 (CD44-pos, CD25-pos), DN3 (CD44-neg, CD25-pos) and DN4 (CD44-neg, CD25-neg) transition stages. Positively selected DP and SP thymocytes were identified by surface CD5 and CD69 upregulation. All populations were quantified as a relative percentage of the parent population.

### Binding of anti-human and anti-mouse CD3 antibodies to hCD3E-epi T cells and T cell activation with plate-bound anti-CD3 antibodies

AF647-conjugated hCD3.1/KLH and m2C11/KLH bispecific antibodies were used to stain RBC-lysed hCD3E-epi splenocytes isolated with gentle MACS tissue dissociator (Miltenyi Biotech), or healthy human PBMC’s isolated from healthy volunteers by leukophoresis (Stemcell Technology; Donor CE0005513, Lot 1,909,423,000, 10/16/2019). Cells were resuspended at 1 × 10^7^ cells/ml in flow buffer, stained with Fc-block, and live/dead fixable dye for 20 min as described above, and pelleted into wells of a 96 well round bottom plate at 1 × 10^6^ cells/well. Cells were stained with a cocktail of fluorescently conjugated antibodies against mouse or human CD19, CD4, and CD8, as well as a fivefold serial dilution of APC-conjugated hCD3.1-KLH and m2C11-KLH antibodies for 30 min at 4 °C. After 2 washes with flow buffer, cells were fixed and acquired on an LSRFortessa X-20 as described above. Staining intensity of the anti-CD3 antibodies on the surface of CD4-pos and CD8-pos T cells was calculated from the mean fluorescence intensity of the APC signal. To measure T cell activation, anti-hCD3.1-KLH was coated onto wells of a 96-well flat bottom plate starting at 10 ug/ml in PBS with 6 × fourfold serial dilutions. Plates were incubated at 4 °C overnight and washed 3 times with RPMI (Corning) containing 10% HI-FBS. Splenocytes from WT C57BL/6 and hCD3E-epi mice, and human PBMC’s were prepared as above, and 200 ul of cells at 5 × 10^6^ cells/ml were added to pre-coated wells and incubated at 37 °C and 5% CO_2_ for 72 h. Following stimulation, cells were pelleted into 96-well round bottom plates, incubated with Fc-block and live/dead fixable dye, stained with fluorescently conjugated antibodies against mouse or human CD4, CD8, CD19, CD25, and CD69, and fixed prior to analysis as decribed above. Cells were acquired and analyzed as above, and T cell activation was measured as the percentage of CD8-positive T cells that upregulated surface CD25 or CD69.

### In vitro T cell-redirected killing of CD20-positive B cells

RBC-lysed single cell suspensions of splenocytes from WT C57BL/6 and hCD3E-epi mice were prepared as described above and resuspended in RPMI + 10% HI-FBS. 100 µl of total splenocytes at 1 × 10^7^ cells/ml were transferred to wells of a 96 well round bottom plate and mixed with a 2 × solution (starting at 20 µg/ml) of fourfold serially diluted CD3-bispecific antibodies (hCD3.1-KLH, hCD3.1-mCD20, m2C11-KLH, m2C11-mCD20). Cells were incubated at 37 °C and 5% CO_2_ for 72 h prior to measurement of B cell depletion by flow cytometry. Cells were pelleted and pre-incubated with Fc-block and live-dead fixable dye, followed by staining with fluorescently conjugates antibodies against CD4, CD8, CD19, CD25, and CD69 and cytofix treatment as described above. Cells were acquired with a BD LSRFortessa X-20 in plate-mode, with settings adjusted so the same volume of sample would be acquired for every well. Only live cells were included in the final analysis with FlowJo V10.6.2. The absolute number of live CD19-positive B cells acquired was used to calculate the percentage of B cell killing using the following equation: % B cell killing = [(1-B cell number in treatment condition)/B cell number in no treatment control]*100. T cell activation was measured in the same samples by measuring CD25 and CD69 surface upregulation on CD8-pos T cells as described above.

### In vivo B cell depletion

WT C57BL/6 (n = 4) and hCD3E-epi mice (n = 4) were pre-bled submentally (30 µl into EDTA tubes) to obtain pre-treatment peripheral B cell frequencies, and then treated the following day intravenously with 1 mg/kg of CD3-bispecific antibodies (hCD3.1-KLH, hCD3.1-mCD20, m2C11-KLH, and m2C11-mCD20) or 10 mg/kg mCD20-mG2a. 30 µl of whole blood was collected by submental method from all mice into EDTA tubes on day 1, 3, and 7 post-treatment. Blood samples were cleared of red blood cells with ACK lysing solution (Biolegend) and stained as described above with fluorescently-conjugated antibodies against mouse CD45, CD19, CD4, CD8, CD25, and CD69. The percentage of CD19-positive B cells remaining in the blood was calculated as a frequency of total live CD45-poitive cells, and T cell activation was measured as the percentage of CD8-positive T cells expressing surface CD25 or CD69. Similar analyses were performed on RBC-lysed splenocytes from mice on day 7 after treatment to measure B cell depletion in secondary lymphoid organs.

### Antibodies for flow cytometry

anti-mouse CD4, fluor PE-CF954, clone RM4-5, cat# 562,285, lot# 9,291,022, vendor BD.

anti-mouse CD8-alpha, fluor APC-H7, clone 53–6.7, cat# 560,182, lot# 834,666, vendor BD.

anti-mouse CD45R/B220, fluor V500, clone RA3-6B2, cat# 561,226, lot# 9,066,743, vendor BD.

anti-mouse CD25, fluor FITC, clone 7D4, cat# 553,072, lot# 9,073,690, vendor BD.

anti-mouse CD44, fluor PE-Cy7, clone IM7, cat# 560,569, lot# 9,102,739, vendor BD.

anti-mouse TCR-beta, fluor APC, clone H57-597, cat# 533,174, lot# 9,014,534, vendor BD.

anti-mouse CD5, fluor V450, clone 53–7.3, cat# 561,244, lot# 8,333,613, vendor BD.

anti-mouse CD69, fluor PE, clone H1.2F3, cat# 553,237, lot# 9,274,837, vendor BD.

anti-human CD4, fluor FITC, clone SK3, cat# 566,320, lot# 9,128,980, vendor BD.

anti-human CD8 fluor APC, clone SK1, cat# 980,904, lot# B328671, vendor Biolegend.

anti-human CD19, fluor BV650, clone HIB19, cat# 302,238, lot# B300887, vendor Biolegend.

anti-human CD69 fluor PE-Cy7, clone FN50, cat# 310,912, lot# B259693, vendor Biolegend.

anti-human CD25, fluor BV421, clone BC96, cat# 302,630, lot# B290363, vendor Biolegend.

### Protein expression and purification

Human or murine chimeric Fabs were individually cloned into the pTT5 expression plasmids (GenScript) with an N-terminal osteonectin signal peptide and a C-terminal poly-histidine tag on the heavy chain. Supplemental Table S1 lists the VH and VL sequences. The Fabs were transiently expressed in Expi293 cells (ThermoFisher Scientific), and the clarified supernatants were purified using Ni Sepharose excel resin (GE Healthcare Life Sciences) followed by a size exclusion purification step using a Superdex 200 column (GE Healthcare Life Sciences) into 1 × PBS.

Human CD3E/D, cyno CD3E/D, murine CD3E/D, and chimeric human/murine CD3E/D were generated as Fc-fused heterodimers, and similarly cloned into pTT5 expression plasmids. Each heterodimer was transiently co-expressed in Expi293 cells. Clarified supernatants were purified using MabSelect Sure LX resin (GE Healthcare), washed with PBS, eluted in 100 mM pH 3.6 sodium citrate buffer, and neutralized with 1 M pH 8.0 Tris buffer. Each CD3 heterodimer was further purified on a Superdex 200 column into 1 × PBS.

hCD3.1/CD20 mG1 D265A, hCD3.1/Neg mG1 D265A, 2C11/CD20 mG1 D265A, and 2C11/Neg mG1 D265A bispecific antibodies were generated as previously described^7^. Briefly, each bispecific was expressed by the simultaneous transfection of four plasmids each encoding different polypeptide chains (HC1, HC2, LC1, and LC2) into Expi293 cells. HCs and LCs were transfected at optimal DNA ratios. The supernatant was purified using MabSelect Sure LX (GE Healthcare) columns, respectively, washed with PBS, eluted in 100 mM pH 3.6 sodium citrate buffer, neutralized with 1 M pH 8.0 Tris buffer, and dialyzed into PBS. Isolated protein was next diluted 5 × into 25 mM pH 5.5 MES buffer, and further purified by gradient elution from a cation exchange column. Bispecifics were isolated, buffer exchanged into 1 × PBS, and further characterized by analytical SEC (Agilent Technologies 1260), LC/MS (Agilent Technologies 1290 Infinity/ Agilent Technologies 6530 Accurate-Mass Q-TOF), and SDS PAGE (Fig. S6).

### HDX-MS approach for epitope characterization

HDX-MS was conducted as described previously^24^. In brief, non-deuterated experiments were performed to generate a list of common peptides for CD3 prior to epitope mapping experiments. For epitope mapping in CD3, samples including CD3 alone and protein complex of mAb/CD3 were prepared at 1 to 1 molar ratios at 20 µM final protein concentration in PBS at 25 °C for 1 h prior to HDX. HDX was conducted on an HDX PAL robot (LEAP Technologies, Carrboro, NC) where 5 µL of each sample was diluted into 55 µL of D_2_O buffer (10 mM phosphate buffer, D2O, pD 7.0) and the reactions were carried out for different periods of time: 20 s, 1 min, 10 min and 240 min. By the end of each labeling reaction period, the reaction was quenched by adding quenching buffer (100 mM phosphate, 4 M GdmCl, 0.4 M TCEP, pH 2.5, 1:1, v/v) and 50 µL of quenched sample was injected into Waters nanoACQUITY UPLC HDX Manager™.

Proteins were digested online using a Enzymate pepsin column, 300 Å, 5 µm, 2.1 mm × 30 mm (Waters Corp., Milford, MA, USA), at 20 °C and for 3 min. The deuterated peptides were trapped and desalted for 3 min on an ACQUITY UPLC BEH C18 VanGuard Pre-column (130 Å, 1.7 µm, 2.1 mm × 5 mm). Peptides were separated with an ACQUITY UPLC C18 BEH 1.0 × 100.0 mm column (Waters Corp.) with a 10 min gradient of 8–85% acetonitrile/water containing 0.1% formic acid operated under 40 µL/min flow rate. All the chromatographic elements were held at 0.0 ± 0.1 °C. Mass spectra were obtained with a Waters Synapt G2si Q-TOF equipped with standard ESI source (Waters Corp.). The instrument configuration was the following: capillary was 3.5 kV, sampling cone at 35 V, source temperature of 80 °C and desolvation temperature of 175 °C. Mass spectra were acquired over an m/z range of 260 to 2000.

For HDX-MS data analysis, peptic peptides were identified through a combination of exact mass analysis and MSE using ProteinLynx Global Server 3.0.2 (Waters Corp., Milford, MA, USA). Peptide-level deuterium uptakes were calculated using Waters DynamX ™ 3.0 software.

### Surface plasmon resonance (SPR) spectroscopic determination of anti-CD3 variants binding parameters

SPR was used to determine binding parameters for the anti-CD3 Fabs to the Fc-tagged human CD3E/D, cyno CD3E/D, murine CD3E/D, and the chimeric human/murine CD3E/D heterodimer variants with a BIACORE® T200 SPR spectrometer (Biacore AB). The CD3 variants were captured on a C1 chip where either anti-human Fc pAb or anti-murine Fc pAb was first amine coupled to the surface. The CD3 variants were then bound by a concentration series of the Fabs in HBS pH 7.4 running buffer supplemented with 0.05% Tween-20, 1 g/L BSA at 37 °C. All data were double-referenced and fitted to a 1:1 Langmuir binding model with mass transport to determine equilibrium dissociation constants (K_D_).

### Research ethics

All animal studies that were conducted are in accordance with the ARRIVE guidelines and are in compliance with relevant ethical regulations. Animal studies performed at Bristol-Myers Squibb were carried out in an AAALAC, Intl-accredited facility and approved by the Bristol-Myers Squibb Institutional Care and Animal Use Committee.

## Supplementary Information

Supplementary Information.
