# Supplementary material for: Humanization of a strategic CD3 epitope enables evaluation of clinical T-cell engagers in a fully immunocompetent in vivo model
Source: Sci Rep. 2022 Mar 3;12:3530. doi: 10.1038/s41598-022-06953-7 (PMC8894342; doi:10.1038/s41598-022-06953-7)
Supplement: Supplementary file 1 — Supplementary Information. [file 41598_2022_6953_MOESM1_ESM.pdf]

SUPPLEMENTAL DATA

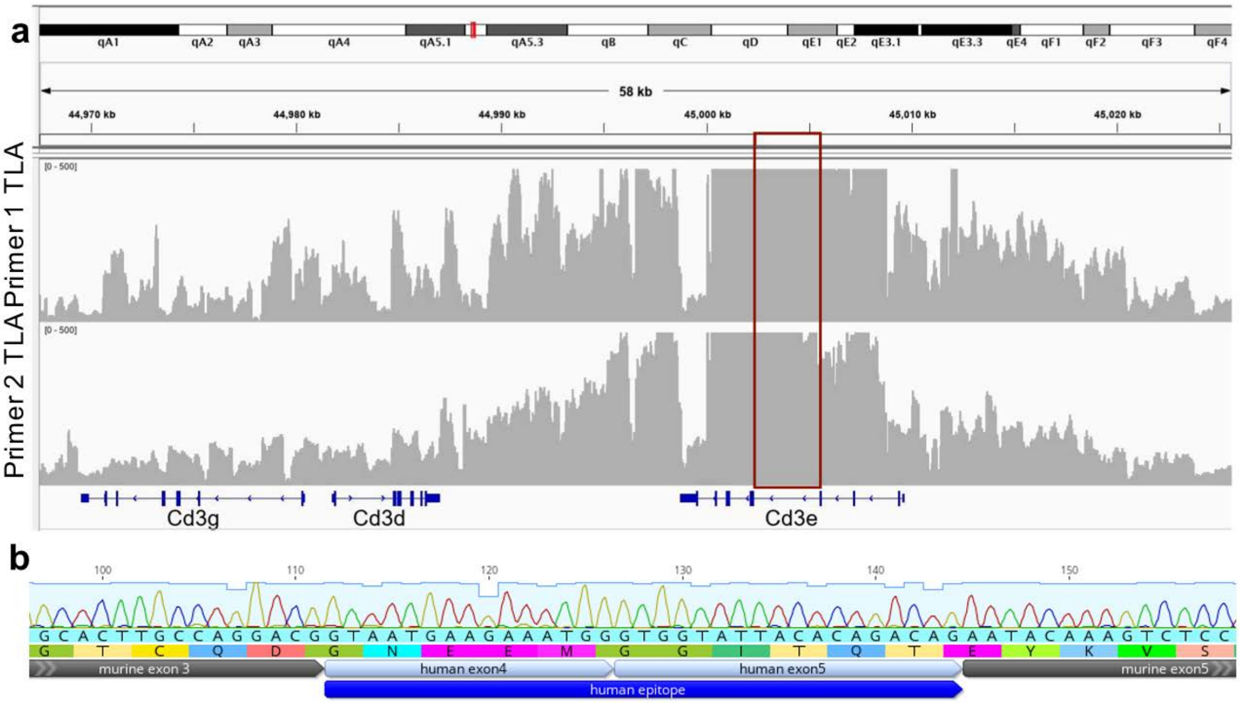

Figure S1. Sequence characterization of hCD3E-epi mice. (A) Splenocytes of the hCD3E-epi mice were collected and DNA was purified for TLA sequencing analysis. Reads were mapped back to the humanized sequence of the CD3e gene. Coverage across the humanized CD3e gene and wildtype CD3d and CD3g locus is shown in grey across different sequence amplifiers with one located within the humanized region and one located outside the humanized region in murine germline sequence. Humanized region marked in red box. Sequencing reads are contiguous across the humanized Cd3e locus and across the neighboring Cd3d and Cd3g locus without unexpected rearrangements. (B) Sanger sequencing of endpoint RT-PCR of T cells purified from the thymus of 20-week old mice across the humanized region of the CD3E-epi mice. Neighboring exons and humanized epitope are annotated below the confirmed reference sequence.

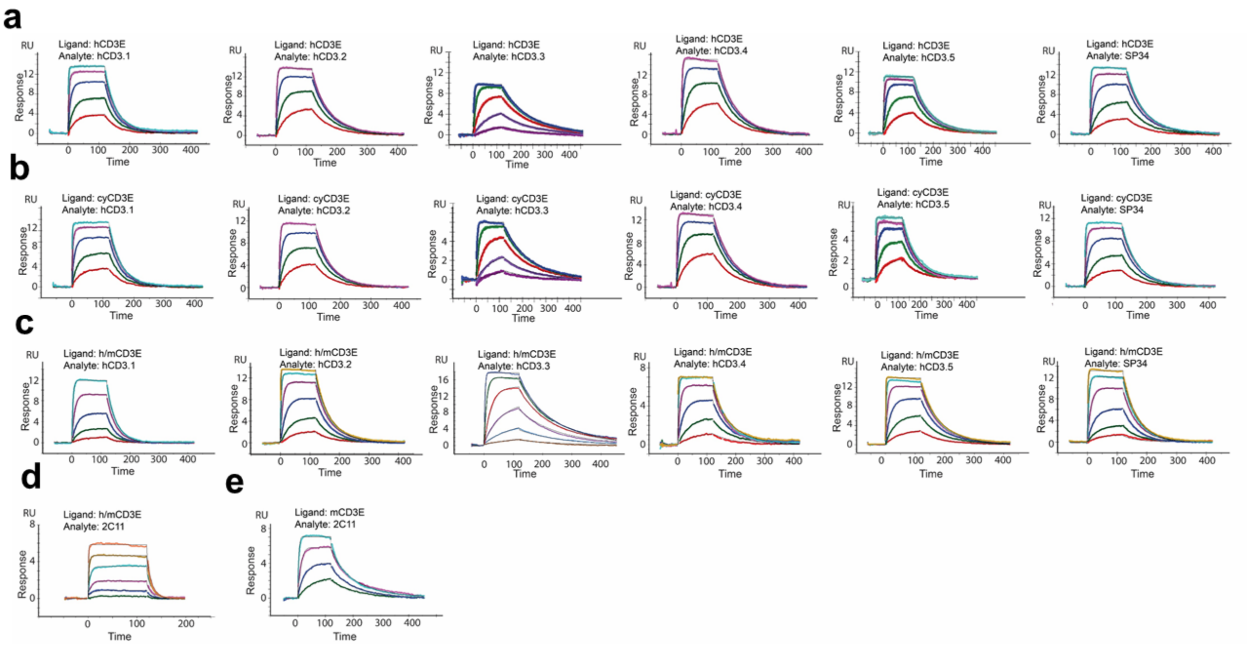

Figure S2. **Binding of anti-CD3 Fabs to CD3ε/δ variants.** SPR binding signals of Fabs to immobilized (A) mFc-tagged human CD3ε/δ, (B) mFc-tagged cyno CD3ε/δ, (C-D) hFc-tagged human/murine chimeric CD3ε/δ, and (E) hFc-tagged murine CD3ε/δ. The signals shown in (A-B) use Fab concentrations corresponding to the following color code: 200 (cyan), 66.7 (light purple), 22.2 (blue), 7.4 (green), 2.5 (red), 0.82 (purple), and 0.27 (dark purple) nM. The data in (C) includes the following Fab concentrations: 1000 (yellow), 333 (cyan), 111 (light purple), 37 (blue), 12.3 (green), 4.1 (red), 1.37 (purple), 0.46 (light blue), and 0.15 (brown) nM. The data in (D-E) includes the following Fab concentrations: 3000 (orange), 1000 (yellow), 333 (cyan), 111 (light purple), 37 (blue), 12.3 (green) and 4.1 (red) nM.

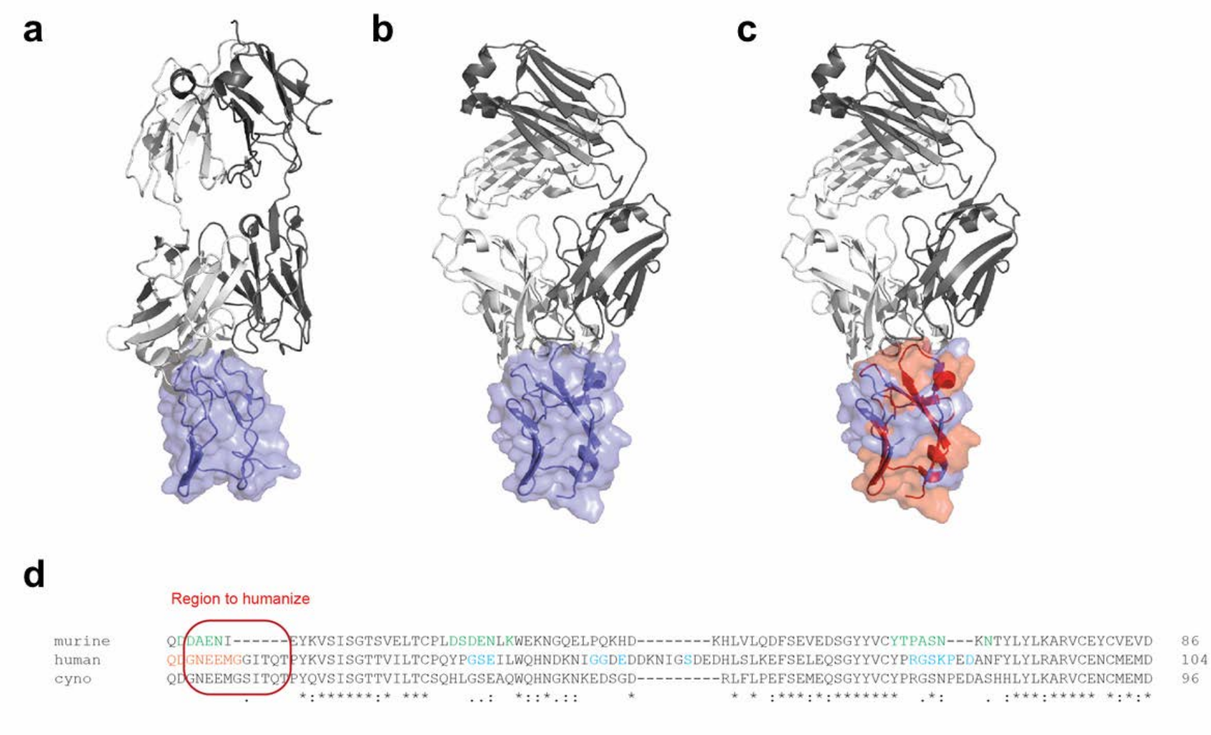

Figure S3. Characterization of the 2C11 and OKT3 epitopes based on structural data. (A) 2C11 in complex with murine CD3ε (PDB ID 3R08). (B) OKT3 in complex with human CD3 ε/δ (PDB ID 1SY6). Only CD3ε is shown. Human and murine CD3ε were aligned, and map to a similar region on CD3. (C) OKT3 in complex with human CD3ε/δ (PDB ID 1SY6) with residues conserved between human and cyno highlighted in red. OKT3 epitope maps to highly conserved region between human and cyno CD3ε. (D) A sequence alignment of human, murine, and cyno CD3ε. The contact residues for hCD3.1-5 (based on HDX) on highlighted in orange. The contact residues for 2C11 (based on the crystal structure) are highlighted in green. The contact residues for OKT3 (based on the crystal structure) are highlighted in blue.

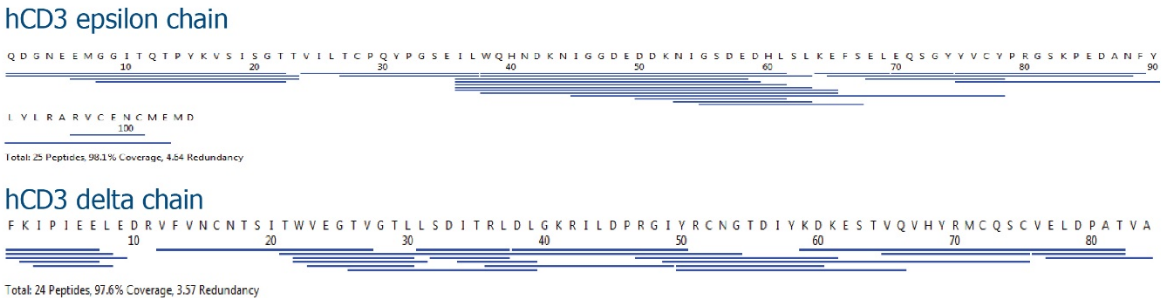

Figure S4. HDX-MS hCD3ε chain sequence coverage.

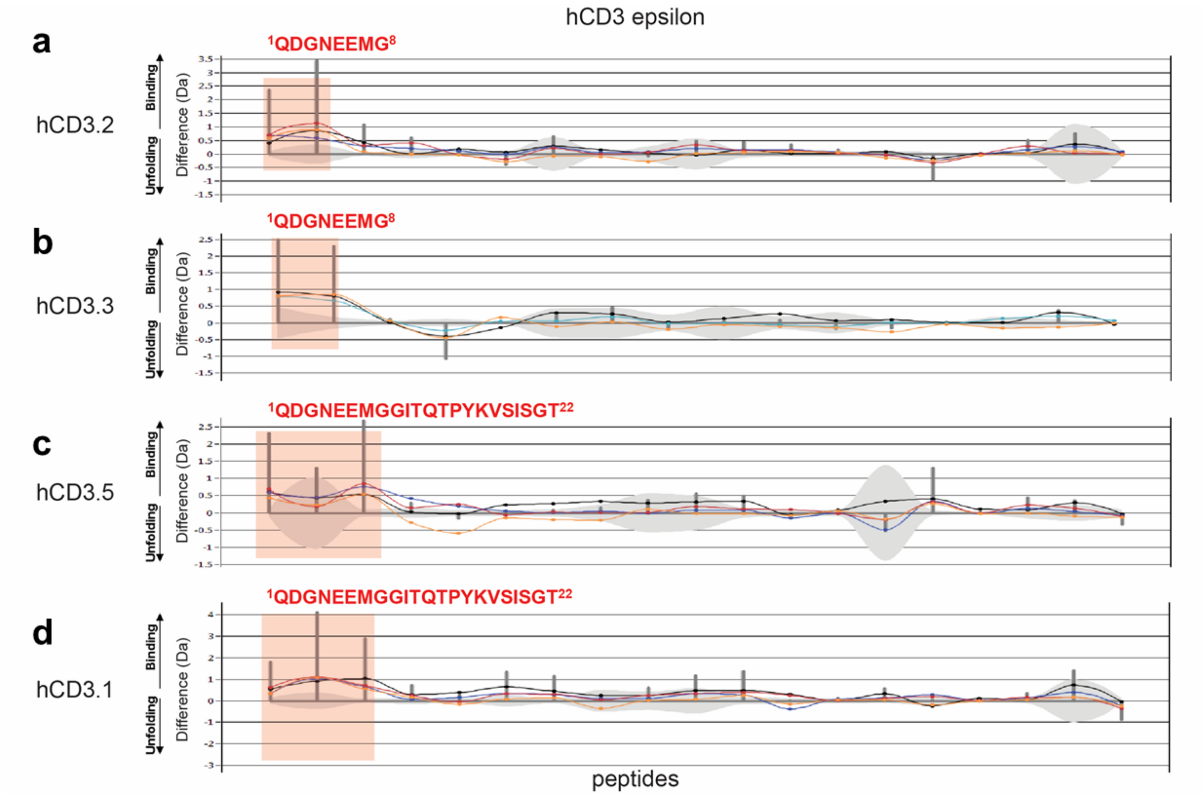

Figure S5. Differential HDX of the CD3 ε-subunit in CD3 vs. mAb/CD3 complex. Deuterium uptake differences across four exchange time points, 20 sec (yellow), 1 min (red), 10 min (blue) and 240 min (black), were summed and shown as the gray bars. Error band with 1σ is shown in gray. Red box indicates the primary epitope of each antibody.

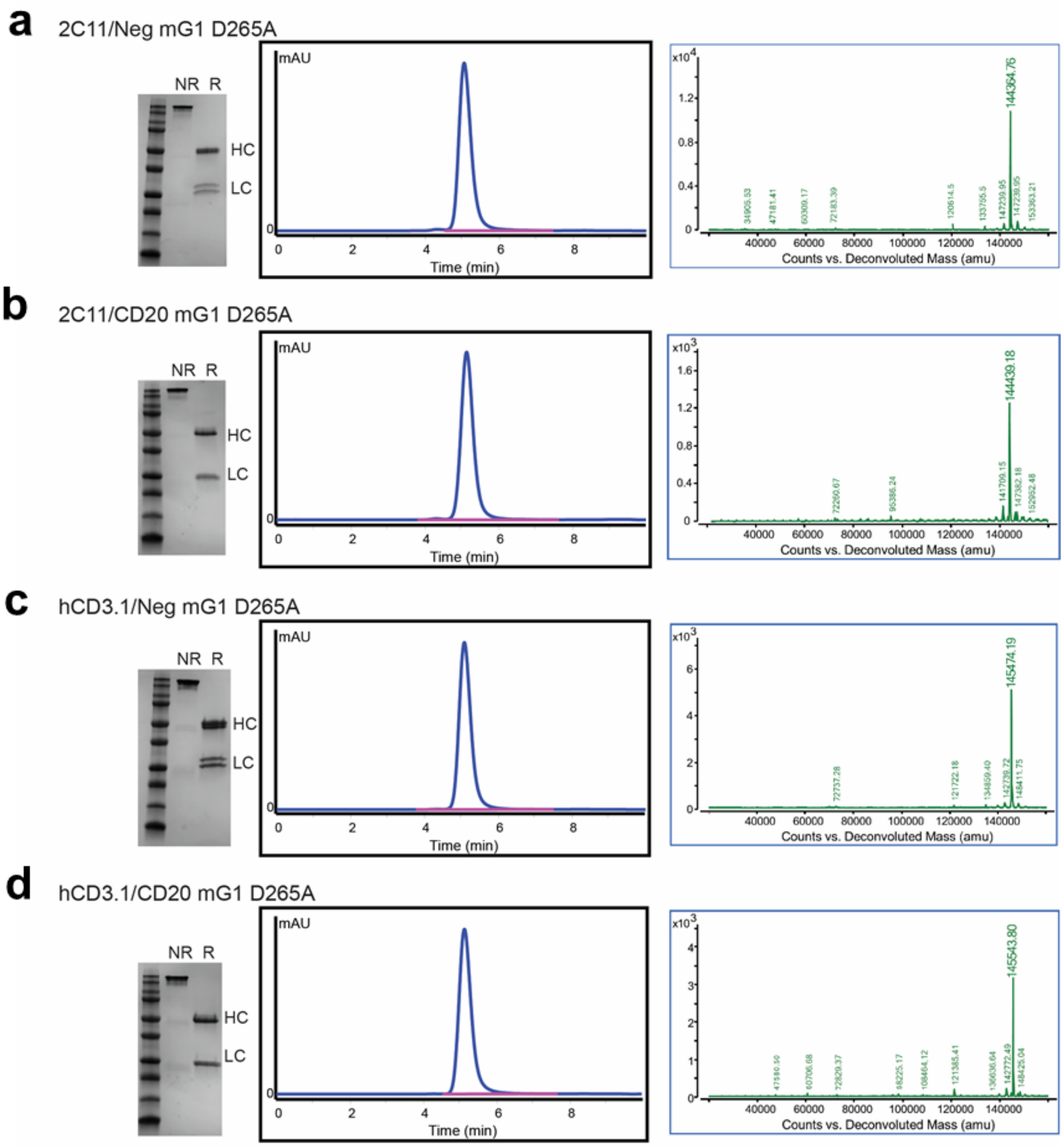

Figure S6. Analytical characterization of bispecifics. Non-reducing (NR) and reducing (R) SDS-PAGE, analytical-SEC, and LC/MS confirmed the purity and identity of (A) 2C11/Neg mG1 D265A, (B) 2C11/CD20 mG1 D265A, (C) hCD3.1/Neg mG1 D265A, and (D) hCD3.1/CD20 mG1 D265A.

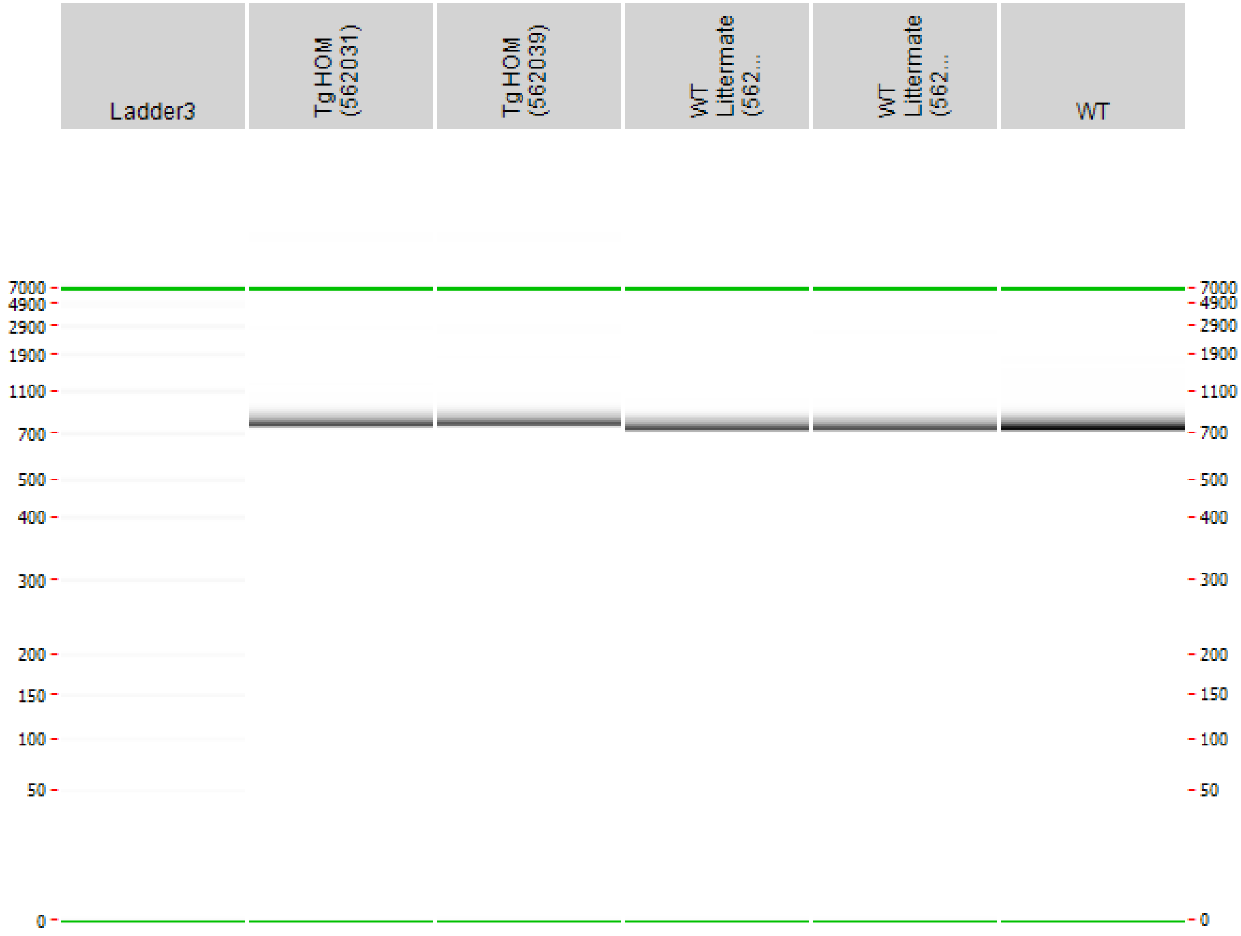

Supplemental FigureS7: Full length uncropped gel from Figure 2 Panel B.

Anti-CD3 variant	**VH**	**VL**
hCD3.1	EVQLVESGGGLVQPGGSLRLSCAASG<br>FTFSDYYMTWVRQAPGKGLEWVAFIR<br>NRARGYTSDHNPSVKGRFTISRDNAK<br>NSLYLQMNSLRAEDTAVYYCARDRPS<br>YYVLDYWGQGTTVTVSS	DIVMTQSPDSLAVSLGERATINCKSSQSL<br>FNVRSRKNYLAWYQQKPGQPPKLLISWA<br>STRESGVPDRFSGSGSGTDFTLTISSLQA<br>EDVAVYYCKQSYDLFTFGSGTKLEIK
hCD3.2	EVQLVESGGGLVQPGGSLRLSCAASG<br>FTFNTYAMNWVRQAPGKGLEWVARIR<br>SKYNNYATYYAASVKGRFTISRDDSKN<br>SLYLQMNSLKTEDTAVYYCARHGNFG<br>NSYVSWFAYWGQGTLVTVSS	QTVVTQEPSLTVSPGGTVTLTCRSSTGA<br>VTTSNYANWVQQKPGQAPRGLIGGTNKR<br>APGTPARFSGSLLGGKAALTLSGVQPED<br>EAEYYCALWYSNLWVFGGGTKLTVL
hCD3.3	EVQLVESGGGLVQPGGSLKLSCAASG<br>FTFNKYAMNWVRQAPGKGLEWVARIR<br>SKYNNYATYYADSVKDRFTISRDDSKN<br>TAYLQMNNLKTEDTAVYYCVRHGNFG<br>NSYISYWAYWGQGTLVTVSS	QTVVTQEPSLTVSPGGTVTLTCGSSTGA<br>VTSGNYPNWVQQKPGQAPRGLIGGTKFL<br>APGTPARFSGSLLGGKAALTLSGVQPED<br>EAEYYCVLWYSNRWVFGGGTKLTVL
hCD3.4	QVQLVQSGAEVKKPGASVKVSCKASG<br>FNIKDYYMHWVRQAPGQRLEWMGWI<br>DLENGNTIYDPKFQGRVTITRDTSASTA<br>YMELSSLRSEDTAVYYCARDGYGRYF<br>YDVWGQGTLVTVSS	DIVMTQSPDSLAVSLGERATINCKSSQSL<br>LNSRTGKNYLAWYQQKPGQPPKLLIYWA<br>STRESGVPDRFSGSGSGTDFTLTISSLQA<br>EDVAVYYCKQSYSRRTFGGGTKVEIK
hCD3.5	EVQLLESGGGLVQPGGSLRLSCAASG<br>FTFSTYAMNWVRQAPGKGLEWVSRIR<br>SKYNNYATYYADSVKGRFTISRDDSKN<br>TLYLQMNSLRAEDTAVYYCVRHGNFG<br>NSYVSWFAYWGQGTLVTVSS	QAVVTQEPSLTVSPGGTVTLTCGSSTGA<br>VTTSNYANWVQEKPGQAFRGLIGGTNKR<br>APGTPARFSGSLLGGKAALTLSGAQPED<br>EAEYYCALWYSNLWVFGGGTKLTVL
SP34	EVQLVESGGGLVQPKGSLKLSCAASG<br>FTFNTYAMNWVRQAPGKGLEWVARIR<br>SKYNNYATYYADSVKDRFTISRDDSQS<br>ILYLQMNNLKTEDTAMYYCVRHGNFGN<br>SYVSWFAYWGQGTLVTVSA	QAVVTQESALTTSPGETVTLTCRSSTGAV<br>TTSNYANWVQEKPDHLFTGLIGGTNKRA<br>PGVPARFSGSLIGDKAALTITGAQTEDEAI<br>YCALWYSNLWVFGGGTKLTVL
2C11	EVQLVESGGGLVQPGKSLKLSCEASG<br>FTFSGYGMHWVRQAPGRGLESVAYIT<br>SSSINIKYADAVKGRFTVSRDNAKNLLF<br>LQMNILKSEDTAMYYCARFDWDKNYW<br>GQGTMVTVSS	DIQMTQSPSSLPASLGDRVTINCQASQDI<br>SNYLNWYQQKPGKAPKLLIYYTNKLADG<br>VPSRFSGSGSGRDSSFTISSLESEDIGSY<br>YCQQYYNYPWTFGPGTKLEIK

Supplementary Table S1. Sequence information for anti-CD3 variants.
